# A 44-year-old patient with a new-onset seizure disorder after vaccination against Japanese encephalitis: a case report

**DOI:** 10.1186/1752-1947-7-66

**Published:** 2013-03-12

**Authors:** Christoph Schulze

**Affiliations:** 1 Department of Orthopaedics, University Medicine Rostock, Doberaner Strasse 142, Rostock, 18057, Germany; 2 Bundeswehr Institute of Sports Medicine, Dr.-Rau-Allee 32, Warendorf, 48231, Germany

**Keywords:** Adverse reaction, Japanese encephalitis, Seizure, Vaccination

## Abstract

**Introduction:**

Seizure disorders can have a wide variety of causes. In many cases, however, the underlying cause remains unknown. Vaccinations, for example, can trigger seizures, especially during childhood. In the literature, many cases have been reported in which febrile convulsions occurred after the administration of different types of vaccines, such as the measles, mumps and rubella vaccine or the tetanus and diphtheria vaccine. Only a few cases of epilepsy after vaccination have thus far been described in adults.

**Case presentation:**

In the case reported here, a 44-year-old German Caucasian man working as a soldier had a seizure the day after he received a third dose of Japanese encephalitis vaccine. Before this vaccination, he had received multiple vaccines that he had tolerated well. He underwent several drug therapies at various institutions but has continued to experience different forms of seizures for more than 18 months. The intervals between seizures were approximately six weeks in length. The present work discusses our patient’s history, including all diagnostic procedures and results, as well as treatment approaches. None of the examinations revealed a possible cause for the seizures. Since no structural or genetic causes were detected, the seizures were deemed most likely to have been caused by the vaccinations, especially vaccination against Japanese encephalitis. To date, no medication has prevented our patient from having repeated attacks.

**Conclusions:**

To the best of our knowledge there have been no previous cases reported in the literature where seizures occurred after multiple vaccinations in general or after vaccinations against Japanese encephalitis in particular. Although vaccines are tested before release, the appearance of new adverse reactions cannot be prevented in all cases. Seizure after vaccination is difficult to treat. In our patient’s case, different approaches have not led to a satisfying result to date.

## Introduction

Vaccinations can cause a wide variety of adverse reactions. Since the early days of vaccination, typical vaccine-specific adverse events have been widely reported in the literature. Vaccinations can be associated with technical problems, such as injections at inappropriate sites, or with injuries to a bone, the periosteum, a muscle or other structures [1,2]. They can also result in adverse effects, which can be divided into allergic and severe systemic reactions, including disabilities, death, febrile and non-febrile seizures, encephalitis and/or encephalopathy, sudden infant death syndrome and speech disorders [1,2]. There is also variation in how the incidence of adverse reactions is reported in the literature. For example, the incidence of anaphylactic reactions is estimated to be 0.63 per 1,000,000 people vaccinated after receipt of Japanese encephalitis vaccine (JEV), 0.95 per 1,000,000 people vaccinated after receipt of diphtheria, pertussis and tetanus (DPT) vaccine, and 0.68 per 1,000,000 people vaccinated after receipt of influenza vaccine [3]. The causative component has not yet been identified, but the number of adverse reactions was reduced after gelatin was removed from DPT vaccines [3]. From 1994 to 2004, the incidence of serious neurological problems was 0.1 to 0.2 per 1,000,000 vaccinations in Japan [3]. In a clinical study conducted in the USA that investigated the risk of seizures in patients after receiving DPT and measles, mumps and rubella (MMR) vaccines, the incidence of seizures was reported to be between three and 35 cases per 100,000 people vaccinated, depending on the vaccine used [4]. Seizures attributable to vaccination have been reported after administration of hepatitis B vaccine [5], tick-borne encephalitis (TBE) vaccine [6], MMR, oral polio vaccine, *Hemophilus influenzae* type B (Hib) vaccine and DPT vaccine [7,8]. These reports predominantly describe patients who were vaccinated during childhood. Epilepsy after vaccination has, however, also been reported in adults. Whereas Hartmann [5] presented a pediatric case of febrile convulsions after hepatitis B vaccination, Kaygusuz *et al*. [9] reported the occurrence of non-febrile convulsions in an adult. The risk of seizures exists from the day of vaccination (for DPT) until 14 days after vaccination (for MMR) [4]. Although allergic reactions have been described after receiving JEV [10,11], no case of a seizure attributable to this vaccine has been reported to date. In addition, the information sheet on JEV (JE-VAX®, Aventis Pasteur, Swiftwater, PA, USA) contains no information that would suggest a risk of seizures. In the case reported here, our patient had a seizure after having received a third dose of JEV. Prior to this incident, he had been given multiple pre-deployment vaccinations within a relatively short period of time.

## Case presentation

Our patient, a 44-year-old Caucasian German man working as a soldier, had no known history of neurological disorders or internal diseases apart from a febrile convulsion of unknown origin during childhood, and hypertension that had been treated with a combination of hydrochlorothiazide and lisinopril (Acercomp®, AstraZeneca, Wedel, Germany) during the two months preceding the seizure. Our patient denied any history of drug or alcohol misuse. He had received multiple pre-deployment vaccinations over a brief period of time. These vaccinations were given in addition to a primary immunization series against MMR, DPT, TBE, hepatitis A and hepatitis B, which he had completed the previous year. Our patient received the following vaccinations: first, a rabies vaccine and vaccines against abdominal typhus, yellow fever and meningococcal meningitis were administered. The second rabies vaccine followed one week later. Our patient received his first dose of JEV after a further week, followed by a third rabies vaccine and the second JEV after two weeks. He received his third dose of JEV four weeks after the previous vaccination and had his first seizure one day later. He was admitted to the Department of Neurology at the Hospital of Guestrow where he underwent several examinations. Temporarily elevated blood pressure levels were detected during 24-hour blood pressure monitoring. Magnetic resonance imaging (MRI) results were affected by artifacts. Of note, in his documented medical history during military service (25 years) there had been no craniocerebral injury. No pathological changes were revealed by the results of any of the following investigative procedures: electroencephalography ([EEG]: α-EEG (frequency: 10-Hz base rhythm), no epilepsy), 24-hour electrocardiography (ECG), lumbar puncture (color: clear, white blood cell count: 1, lactate: 1.58mmol/L, protein content: 371mg/L, no IgG), Lyme disease testing, sleep-deprived EEG and a Schellong test. Laboratory study results revealed elevated creatine kinase levels and myoglobin levels. Since this was the first time our patient had experienced a seizure and no pathological changes were detected, no regular medications were given. Three months later, our patient received a dose of TBE vaccine. Later on the same day, he had a second seizure and was again referred to the Neurological Department at the Hospital of Guestrow. Since our patient refused to be admitted to the hospital, no further diagnostic tests were performed. He was subsequently treated as an out-patient in the Department of Neurology at the German Armed Forces Hospital in Hamburg. Four weeks later he reported a further seizure. On the same day, drug therapy was started using 600mg of extended-release valproate, which was later replaced by 100mg/day of topiramate (Janssen-Cilag, Neuss, Germany). Control EEGs (α-EEG, no epilepsy) did not reveal any pathological changes. In addition, our patient was instructed to discontinue physically strenuous work. Ten weeks later he again had a seizure. Laboratory test results showed that the topiramate concentration was below a therapeutic level. On account of its diuretic effect, the hydrochlorothiazide-containing drug (Acercomp®) was considered to be the likely cause of the low topiramate level. For this reason, it was recommended that the drug be discontinued and the topiramate dose be increased to 125mg/day. However, as Acercomp® had been prescribed for the treatment of our patient’s hypertension and had been well tolerated, it was continued; topiramate was increased to 125mg/day. Control EEGs (α-EEG, no epilepsy) did not reveal any pathological findings. Two months later, our patient had a further epileptic seizure. Neither an EEG nor computed tomography scan demonstrated any abnormal changes. The dose of topiramate was increased to 200mg/day. After a further six weeks, the next seizure occurred. For the first time, EEG results demonstrated the presence of epileptiform abnormalities in the frontotemporal region. Topiramate was replaced by 100mg/day of lamotrigine. Further control EEGs revealed no pathological changes. At six and 18 weeks later, our patient had further seizures, even though his lamotrigine dose had been increased to 200mg/day. In order to obtain artifact-free MRI studies, a small piece of metal located subcutaneously was removed. Subsequent MRI scans detected no pathological changes that could explain the patient's recurrent seizures (i.e., there was no abnormal enhancement of contrast medium and there were no abnormal hippocampal structural changes. Incidental findings of a right central semiovale scar and cystic formation in the right frontal area were noted). His lamotrigine dose was increased to 300mg/day.

Six weeks later our patient was vaccinated against influenza. No complications were observed. However, at six and 12 weeks later the patient again experienced seizures. Treatment with levetiracetam (Keppra®, UCB Pharma, Brussels, Belgium) at a dose of 2000 mg/day was initiated by the Department of Neurology at the University of Rostock Hospital. The levetiracetam dose was given in addition to lamotrigine. Further diagnostic tests were performed in the Department of Neurology at the University of Greifswald Hospital. For diagnostic purposes, all medications were discontinued. This, however, did not induce a seizure. All test results were unremarkable. Lamotrigine was then discontinued and the dose of levetiracetam was increased to 3000mg/day. The occurrence of seizures has subsequently reduced to one to three per year since then.

## Discussion

Although many investigations were performed at various institutions, the cause of our patient’s epileptic seizures remains unknown. The close temporal association between the vaccinations and the first and subsequent seizures suggests, however, that the seizures are the result of an adverse reaction to vaccines, for example, to JEV. The intervals between vaccinations and seizures were within the period during which reactions can possibly occur, according to Barlow *et al*. [4]. Although seizure disorders attributable to vaccinations have mainly been reported in children, similar reactions have also been described in adults [9]. The development of chronic neurological diseases or disability as a result of adverse reaction to vaccination is possible, and the disease’s character in our patient’s case matches the character of immunization-caused neurological adverse events [4,12]. The occurrence of seizures can be mediated by the immune system [13]. JEV is a vaccine that can cause a wide variety of complications, including encephalitis [11,14,15]. Seizures secondary to JEV have, however, not been reported to date. In addition, it remains unclear whether the seizures were associated with JEV alone or with JEV in combination with the many other vaccines that were given to our patient over a brief period of time, or whether the seizures were attributable to one of the other vaccines administered during the relevant period. The close temporal relationship between the seizures and the receipt of vaccines derived from neurotropic viruses (for example, TBE, JEV and rabies vaccine) is, however, indisputable. Because the first seizure occurred after JEV administration, the strongest relationship can be seen here. Unfortunately, it was impossible for us to identify the cause of the single febrile seizure that our patient had in his early childhood and to assess whether this seizure may have predisposed our patient to his current condition. In the absence of other causes, and because of the close temporal association between the first seizure and receiving the JEV, the latter can be considered the most likely cause of the seizures. There was no evidence of the vaccine having been incorrectly administered. Earlier doses of TBE vaccine had been well tolerated, and the seizure occurred only after our patient received a booster dose of TBE vaccine. A possible explanation might therefore be central nervous system sensitization.

German military personnel often receive multiple vaccinations over a brief period of time in preparation for deployment. All vaccinations are given in accordance with the relevant regulations, as well with national and international guidelines, and are associated with a low incidence of complications in routine clinical practice. Certain risks, however, cannot be completely ruled out.

## Conclusions

Vaccinations are an effective means of preventing infectious diseases. Despite comprehensive control measures, precise instructions for the application of vaccines and clinical studies, adverse reactions cannot be completely ruled out and can arise from a variety of causes. In the case presented here, our patient’s new-onset seizure disorder was most likely to have been associated with the receipt of Japanese encephalitis vaccine. It is conceivable that this vaccination induced sensitization to vaccines against other neurotropic agents. To the best of our knowledge this is the first report in the literature of a seizure associated with vaccination against Japanese encephalitis. This report demonstrates that treatment proved very difficult in our patient’s case. A successful standard operating procedure cannot therefore be provided. The treatment of this form of seizure is very difficult, and must be planned individually for each patient.

## Consent

Written informed consent was obtained from the patient for publication of this case report and any accompanying images. A copy of the written consent is available for review by the Editor-in-Chief of this journal.

## Competing interests

The author declares that he has no competing interests.
